# Probing the Proton-Loading Site of Cytochrome *C* Oxidase Using Time-Resolved Fourier Transform Infrared Spectroscopy

**DOI:** 10.3390/molecules25153393

**Published:** 2020-07-27

**Authors:** Elena Gorbikova, Sergey A. Samsonov, Ruslan Kalendar

**Affiliations:** 1 Institute of Biotechnology, University of Helsinki, FIN-00014 Helsinki, Finland; dnapcrlab@gmail.com; 2 Faculty of Chemistry, University of Gdansk, 80-308 Gdansk, Poland; sergey.samsonov@ug.edu.pl; 3 Department of Agricultural Sciences, University of Helsinki, FI-00014 Helsinki, Finland

**Keywords:** cytochrome *c* oxidase, proton-loading site, proton transfer, FTIR spectroscopy, D-channel mutants

## Abstract

Crystal structure analyses at atomic resolution and FTIR spectroscopic studies of cytochrome *c* oxidase have yet not revealed protonation or deprotonation of key sites of proton transfer in a time-resolved mode. Here, a sensitive technique to detect protolytic transitions is employed. In this work, probing a proton-loading site of cytochrome *c* oxidase from Paracoccus denitrificans with time-resolved Fourier transform infrared spectroscopy is presented for the first time. For this purpose, variants with single-site mutations of N131V, D124N, and E278Q, the key residues in the D-channel, were studied. The reaction of mutated C*c*O enzymes with oxygen was monitored and analyzed. Seven infrared bands in the “fast” kinetic spectra were found based on the following three requirements: (1) they are present in the “fast” phases of N131V and D124N mutants, (2) they have reciprocal counterparts in the “slow” kinetic spectra in these mutants, and (3) they are absent in “fast” kinetic spectra of the E278Q mutant. Moreover, the double-difference spectra between the first two mutants and E278Q revealed more IR bands that may belong to the proton-loading site protolytic transitions. From these results, it is assumed that several polar residues and/or water molecule cluster(s) share a proton as a proton-loading site. This site can be propionate itself (holding only a fraction of H+), His403, and/or water cluster(s).

## 1. Introduction

**Figure 1:**
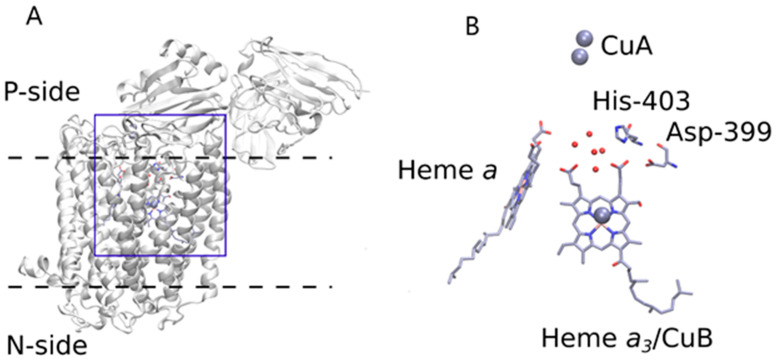
Cytochrome *c* oxidase (C*c*O) overall structure (**A**) and the heme *a_3_* propionates together with located in 5 Å proximity His-403 and Asp-399 and water molecules (red spheres) (**B**). The atomic coordinates are obtained from the structure of *Paracoccus denitrificans* enzyme (PDB ID: 3HB3, 2.25 Å). His-403 and Asp-399 are hydrogen bonded to A-Prp of heme.

Cytochrome *c* oxidase (C*c*O) is the terminal complex (Complex IV) of the respiratory chain of mitochondria, many aerobic bacteria, and archaea. C*c*O contains four redox centers: Cu_A_, heme *a*, heme *a_3_*, and Cu_B_. The last two centers form a binuclear center (BNC) where the reduction of O_2_ to H_2_O takes place. This reaction requires four electrons and four protons (“chemical” protons) to be sent to the BNC. C*c*O accepts electrons from a small soluble enzyme Cytochrome *c* from the outside of the membrane (P-side or positively charged side, see Figure 1) and protons from the matrix of mitochondria or cytosol of bacteria (N-side or negatively charged side, see Figure 1). This reaction is highly exergonic, and energy released is used to translocate four more protons (“pumped” protons) across the membrane (from the N-side to the P-side). Thus, C*c*O takes part in the production of a transmembrane electrochemical gradient (ΔµH^+^) that is used for Adenosine Triphosphate (ATP) synthesis. ATP serves energy-dependent actions in the cell [1,2,3,4].

**Figure 2:**
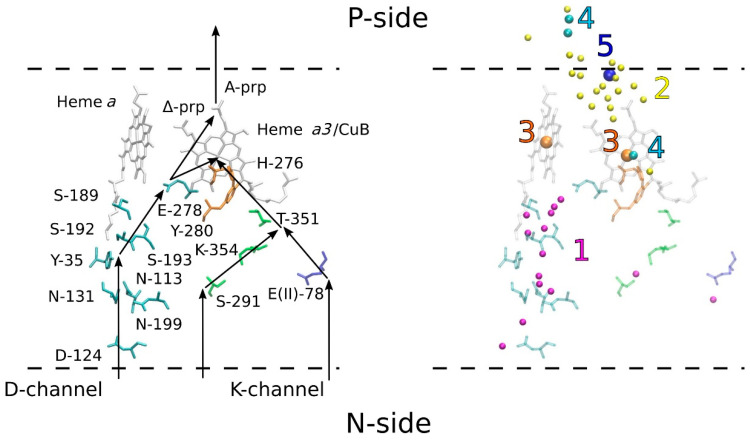
Schematic representation of the proposed proton transfer in C*c*O. Left panel: the proton transfer routes through the D- and K-channels and proton-loading site (PLS) are shown by black arrows. The polar residues composing both channels and hemes without ions are shown in stick representation and labeled. Right panel: water molecules (resolved in the structure within 4.0 Å of these residues and metal ions) and metal ions are shown in spheres and labeled using the following numbers: 1—water molecules within D- and K-channels (magenta), 2—water molecules within PLS (yellow), 3—iron (orange), 4—copper (cyan), 5—manganese (blue). The atomic coordinates are obtained from the structure of *Paracoccus denitrificans* enzyme (PDB ID: 3HB3, 2.25 Å).

Electrons are carried from one redox center to the next by tunneling. In contrast to electrons, protons require special channels filled in with water molecules for their transfer in the protein interior. C*c*O possesses two such entrance channels from the N-side of the membrane, so called D- and K-channels, named after conserved Asp-124 and Lys-354 residues, respectively [5,6,7,8,9,10,11,12]. The current work is concentrated on the D-channel exploiting the mutations of key residues in this channel. The initial proton acceptor in the D-channel is D-124. From this residue, protons pass via a chain of polar residues and water molecules to E-278 (conserved residue in the middle of the membrane), then to Δ-propionate (Prp) of heme *a_3_*, to A-Prp of heme *a_3_* [13,14], and finally to the proton-loading site (PLS or “pump” site) that is located ~1/5 distance from the P-side [15] (Figure 2, reproduced from [16]).

Although the identity of PLS was proposed [17,18,19,20], there was no attempt to directly observe its protonation/deprotonation status. One of the methods that allows protolytic transitions to be shown is Fourier transform infrared (FTIR) spectroscopy, and particularly time-resolved step-scan FTIR (TRS^2^-FTIR) spectroscopy [21,22,23,24,25,26,27,28,29,30,31]. FTIR spectroscopy was successfully applied to cytochrome *c* oxidase (e.g., [27,28]) and many other enzymes such as Ca^2+^ ATPase [32]), light-induced enzymes [33,34], bacteriorhodopsin [35], and redox induced enzymes such as respiratory Complex I [36], cytochrome *bd* [37], and *bc_1_* [38], including time-resolved FTIR studies, for example, on channelrhodopsin-2 [39], bacteriorhodopsin [40,41], photosystem II [42], and cytochrome oxidase *ba_3_* [30,31].

Some mutated D-channel variants of C*c*O (N131V and D124N mutants that were investigated in our previous work) showed enlarged amplitude of the potential generation during the “fast” kinetic phase of the oxygen reaction that was too large to be assigned simply to proton transfer to the BNC from the internal side of the membrane [43,44]. The terms “fast” and “slow” kinetic phases that we widely use throughout the manuscript originate from FTIR kinetic spectra where all the steady-state difference IR spectra comprise the components corresponding to reversible and irreversible processes. When a “slow” process dominates over a “fast” process, the discrepancy between the two respective kinds of difference spectra can be particularly significant. The “fast” components can be resolved only as an abrupt spectral change, while the “slow” component is resolved using a time-resolved FTIR technique. The details on the “fast” and “slow” component for C*c*O are described in more detail in [44]. Because of the fact that the observed amplitude was too high, it was concluded that the proton in these mutated enzymes is loaded to the PLS in the “fast” resolved kinetic phase and dissipates back to the BNC in the following “slow” kinetic phase. This enlarged electrometric amplitude is absent in the E278Q mutant [43]. Since the PLS should be loaded in the aforementioned mutants in the “fast” kinetic phases and unloaded (back to the BNC) in the “slow” kinetic phases, the FTIR time-resolved flow-flash spectra of these two phases should include the PLS protolytic infrared reciprocal bands. The identification of the PLS IR bands assigned to the PLS protonation should be (1) present in N131V and D124N mutants in their “fast” kinetic phases, (2) reciprocal in the “fast” and “slow” phases, with similar position and amplitude, and finally (3) absent in E278Q mutant “fast” kinetic phase. The analysis of these data allowed us to make a suggestion that the “pump” site is not presented by a single entity but is comprised of several contributors. In this manuscript, we assigned the observed IR bands to particular components of PLS. For the first time, probing a proton-loading site of cytochrome *c* oxidase with the use of time-resolved Fourier transform infrared spectroscopy is presented, which opens up a novel experimental procedure to deal with other proton-loading systems, in general.

## 2. Materials and Methods

### 2.1. Setup for Flow-Flash FTIR Measurements

**Figure 3:**
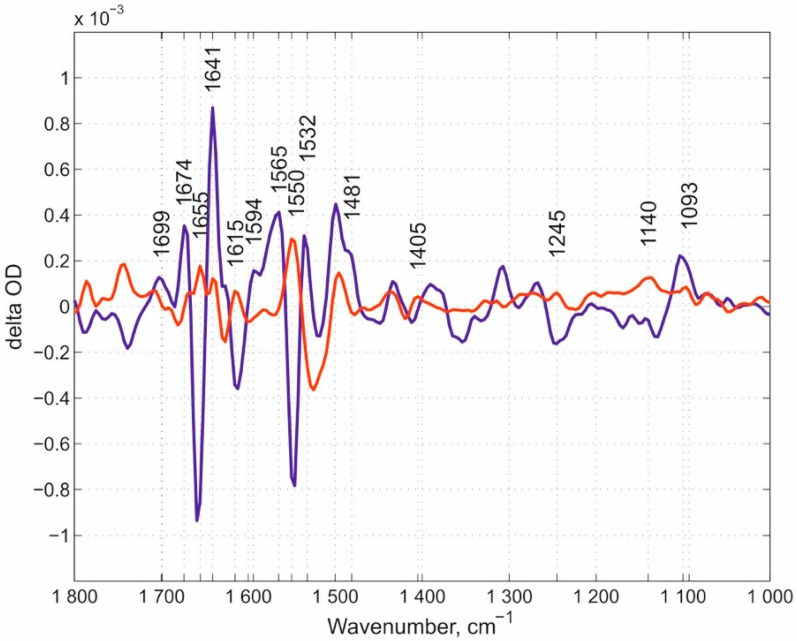
The “fast” and “slow” kinetic spectra of N131V mutant at pH 9.0. The dark-blue spectrum represents the “fast” phase and the red spectrum represents the “slow” one. A total of 14 reciprocal IR bands were found, of about equal position and amplitude in these two kinetic phases. The reciprocal IR bands are marked. Note that in this paper (here and below) the bands that were already earlier assigned, i.e., the Glu-278 band at around 1740 cm^−1^, Tyr-280 at 1308 cm^−1^, Tyr-35 at 1247 cm^−1^, and a shift 1514/1496 cm^−1^, are omitted from consideration.

**Figure 4:**
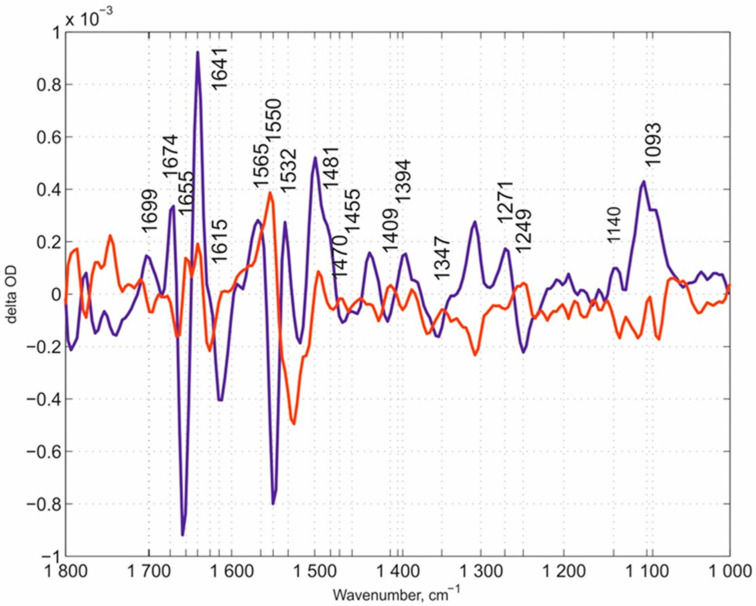
The “fast” and “slow” kinetic spectra of N131V mutant at pH 6.5. The “fast” phase spectrum (in dark blue) and the “slow” one (in red) are shown. Eighteen reciprocal IR bands are marked with numbers.

In order to measure flow-flash reaction (oxygen reaction after photolysis of CO from **FRCO** (fully-reduced CO-inhibited enzyme) complex) on C*c*O, a special setup was assembled [39]. This setup, and the approach itself, were earlier applied to follow the oxygen reaction on C*c*O in real time [44,45,46,47]. The setup is based on an FTIR spectrometer IFS/66s (Bruker Optics, Ettlingen, Germany) equipped with a silicon/ZnSe ATR microprism (SensIR technologies (Chapel Hill, NC, USA), three-bounce version, surface diameter 3 mm) and a fast mercury-cadmium telluride (MCT) detector. The infrared range 1800–1000 cm^−1^ was cut out with an interference filter (Northumbria Optical Coatings Ltd., Boldon, UK). A special homemade chamber was placed on the ATR microprism. For the design of the chamber see [45], Figure 2 therein, and [16], Figure 3 therein. A syringe needle was directed to the center of an ATR sample at a very close distance (0.5 mm) from the sample film to produce high O_2_ concentration for the period of the kinetic experiment. O_2_-saturated buffer was injected with the help of a syringe pump: 100 µL of oxygenated buffer is injected at a speed of 10 mL/min. To initiate the oxygen reaction in **FRCO** inhibited enzyme, the light guide from the laser was mounted into the chamber and faced onto the microprism with the protein film. To follow the C*c*O oxygen reaction in the visible range, a reflectance probe from a visible spectrophotometer HR 2000+ (Ocean optics, St Petersburg, FL, USA) was mounted into the ATR chamber and positioned close to the C*c*O film. A flow-pump is necessary to remove oxygenated buffer from the sample area after measurements of the oxygen reaction. The flow pump was connected to the chamber and switched on in order to accelerate cycles of reduction and CO inhibition of C*c*O. For this purpose, the chamber was connected to a bottle with a “working” buffer that allowed the enzyme to be kept in the **FRCO** state. The whole sequence of events needed to measure oxygen reaction using FTIR and visible reflectance spectroscopies was controlled using a timing board. The sequence of commands from the timing board to the setup can be found in [16], Figure 4 therein.

### 2.2. Enzyme Preparation

Site-directed mutagenesis, bacterial growth conditions, and purification of all three here studied mutated C*c*O enzymes were as described in [48]. The samples were depleted of detergent to be able to immobilize them on the surface of a hydrophobic silicon ATR microprism mainly as described in [49] with modifications for the D124N mutant [45]. After the enzyme was “ATR-ready”, it was positioned on the ATR microprism, dried with a gentle N_2_ flow or under a tungsten fiber lamp (380–1700 nm), and then closed with a specially designed chamber (see the previous section). In the pumping buffer (2 mM potassium phosphate at pH 6.0), the dissolved gases were replaced with 100% CO in the gas-exchange vacuum line. The “working” buffer composition for N131V, D124N, and E278Q mutants can be found in [47] for N131V and [45] for D124N and E278Q. The **FRCO** form of enzymes was prepared as first described in [45]. Briefly, the “working” buffer (100 mM glucose, 260 µg/mL catalase, 3.3 mM ascorbate and 10–100 µM hexaammineruthenium(III) chloride, saturated with pure CO) was pumped over the enzyme film until the enzyme was transformed into the **FRCO** form. This transformation was controlled by the appearance of a C≡O-heme *a_3_* stretching vibration band at 1965 cm^−1^ and took normally between 40 and 80 min.

### 2.3. Sample Quality Control

The quality of C*c*O samples, desired for the flow-flash experiments, was controlled using three different approaches: (1) the appearance, amplitude, and the shape of the heme *a_3_*-C≡O band at 1965 cm^−1^; (2) CO recombination with heme *a_3_* after photolysis of **FRCO** complex, followed using a visible spectrophotometer HR2000+ (Ocean Optics, St Petersburg, FL, USA) (410–700 nm, with 1 ms temporal resolution, kinetics acquisition was triggered by the laser flash); (3) following the CO dissociation in the dark from heme *a_3_*, which was detected using the rapid-scan mode in the region around 1965 cm^−1^; and (4) following a flow-flash reaction in the region of around 1965 cm^−1^ to determine the amount of enzyme capable to perform the oxygen reaction. This region in (3) and (4) was cut out with an interference filter, and the FTIR rapid-scan (RS) mode with time-resolution ~68 ms was applied. The latest approach was performed in the beginning, in the middle, and at the end of each series of flow-flash FTIR kinetic surfaces collection and used to estimate the concentration of catalytically active enzyme (for more details, see [45]). The concentration of active C*c*O was estimated based on the intensity of the 1965 cm^−1^ band [50].

### 2.4. The Oxygen Reaction Measured by Time-Resolved ATR-FTIR Spectroscopy

The oxygen reaction was measured using FTIR time-resolved spectroscopy on three D-channel mutants. All had slower kinetics that allowed measuring the oxygen reaction on them with the RS mode with ~46 ms temporal resolution (i.e., the slow parts of the reaction). The desired spectral region of 1800–1000 cm^−1^ was cut off with an interference filter. The spectral resolution was set at 8 cm^−1^. A background (BG) in the IR region of 1024 coadditions was taken before measuring the oxygen reaction. The apodization function used in Fourier transformation was set at Blackman–Harris 3-term. First, the oxygenated buffer was injected, and, together with it, rapid-scan acquisition was started, followed by a 3 s delay, and, finally, the laser flash fired. Four hundred FTIR spectra were collected, which corresponded to ~18.4 s. The flow-pump was stopped before a BG spectrum was collected and again switched on after the kinetics collection finished. The pump was switched on in order to speed up the C*c*O reduction and **FRCO** complex formation. This cycle was repeated for a number of hours on the same sample film (the enzyme was active during several days of measurements) in order to decrease the ratio signal/noise. All experiments were performed in ice-cooled conditions (0 °C), which slowed down the speed of the oxygen reactions and increased the local O_2_ concentration up to 2.4 mM. Applying the RS FTIR approach, it was possible to resolve kinetically only “slow” kinetic phases of the reaction of mutated proteins with oxygen. Reactions faster than 46 ms were detected only as a spectral jump after the laser flash. To resolve slow phases, a global fitting procedure using three sequential reactions in *Matlab* software (The MathWorks, Inc., Natick, MA, USA) was applied with the SPLMOD algorithm (see [45] for more details). This way, the “slow” kinetic phase in each of the studied mutant enzymes was extracted. Unresolved fast reactions were calculated as a difference between the average of several time points before laser fire and the spectrum measured immediately after the laser flash. These kinetic surfaces were called “fast” kinetic phases.

### 2.5. Data Analysis and Structural Visualization.

The data analysis and spectral data representation were performed with the *Matlab* software. Molecular structures were visualized with Visual Molecular Dynamics Software (VMD, http://www.ks.uiuc.edu/Research/vmd/) [51].

## 3. Results

### 3.1. FTIR Spectra of “Fast” and “Slow” Kinetic Phases of C*c*O Mutants with Blocked D-Channel

**Figure 5:**
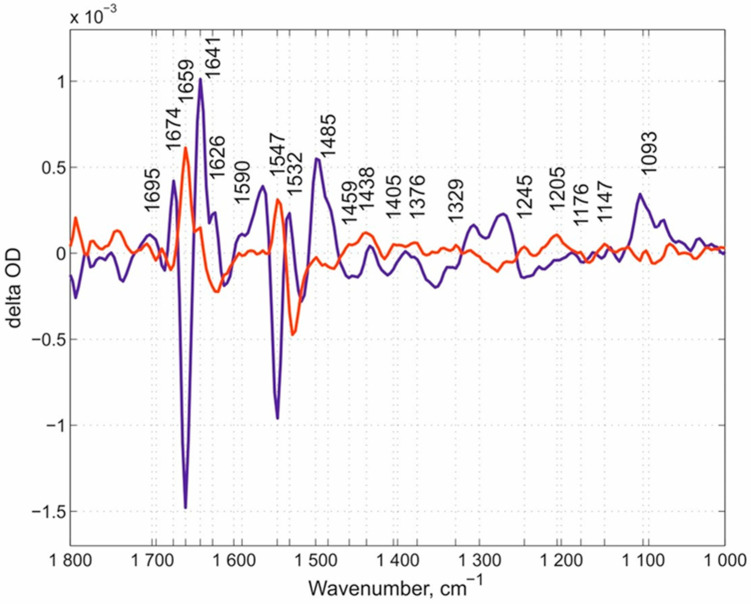
The “fast” and “slow” kinetic spectra of the D124N mutant at pH 9.0. The “fast” kinetic spectrum in dark blue and the “slow” one in red are shown. The reciprocal IR bands are marked with numbers.

Our overview of spectral features begins with the N131V mutant measured at pH 9.0 (Figure 3). Here, we would like to note that we used the pH values in the experiments that rationally address the potential role of each of the mutated residues in the whole proton transfer process and allow for the obtainment of the corresponding spectra. The oxygen reaction in this case, and in all following experiments, was performed in ice-cooled conditions to slow down the reaction rates of mutated enzymes. Using this approach, we managed to resolve the flow-flash reaction using the RS FTIR approach. FTIR kinetic surfaces (Δ optical density-time-wavenumber) (altogether 218 co-additions) were decomposed into two kinetic phases. The first phase (the “fast” kinetic phase) is simply the increase after the laser flash and includes all reactions faster than ~46 ms (time resolution of our RS FTIR method applied). The first resolved phase (we call it here the “slow” kinetic phase) developed with τ ~ 600 ms. The “slow” kinetic phase was nicely fitted with a single exponential equation, which proved that the enzyme population turning over after the laser flash is homogeneous. **FRCO** FTIR photolysis spectra were measured separately (on the wild-type (WT) enzyme at pH 6.5) using continuous mode FTIR spectroscopy with a spectral resolution of 8 cm^−1^ and subtracted from each “fast” phase transition in this case and in all further experiments mentioned in this work irrespectively of the pH value, as it was demonstrated that **FRCO** photolysis (on WT) shows no difference irrespectively of the pH range measured [52]. Such subtraction allowed for a better representation of the differences between the spectra of the mutants and WT enzyme. All spectra in this work were normalized to 1 mM concentration by amplitude of the heme *a_3_*-C≡O band [50] that was photolyzed by a laser flash. The next set of the same data is for the N131V mutant but at pH 6.5: 338 kinetic surfaces were collected altogether. All conditions were essentially the same as for the N131V mutant at pH 9.0. The “slow” kinetic phase was resolved with τ ~ 90 ms (Figure 4), hence the larger noise level relative to N131V at pH 9.0. Here, 18 reciprocal IR bands were found and labeled. The reciprocal IR bands that are present in all three above stated cases are ~1699, ~1674, ~1655, 1641, ~1550, 1532, ~1481, ~1405, ~1245, ~1140, and 1093 cm^−1^, 11 IR bands altogether (the assignments of the spectra mentioned in the manuscript are summarized in Table 1). These bands are further compared with those found in E278Q “fast” kinetic spectra. Figure 5 shows mutated enzyme D124N with 296 kinetic surfaces altogether (conditions are essentially the same as for the previous mutant). In this mutant, 19 reciprocal bands were found.

### 3.2. Comparison of “Fast” Kinetic Spectra of D-Blocked Channel Mutants and E278Q

**Figure 6:**
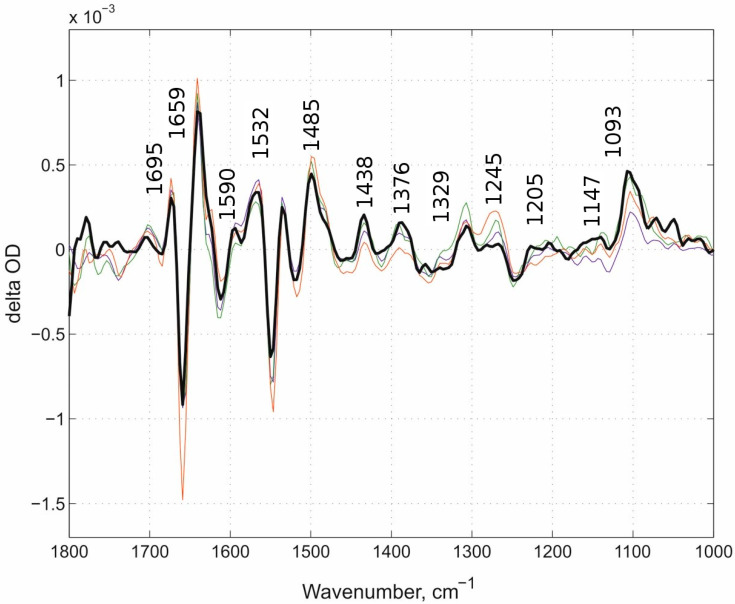
The “fast” kinetic spectra of all three cases, N131V pH 9.0, N131V pH 6.5, and D124N pH 9.0, and of E278Q mutant protein at pH 6.5. The “fast” kinetic spectra of all three cases described above (thin lines of different color) together with the “fast” kinetic spectrum of the E278Q mutant (thick black line).

As stated above, the IR bands that belong to PLS protonation should be absent in the “fast” phase spectrum of the E278Q mutant (Figure 6, spectrum of E278Q was extracted from 118 kinetic surfaces). For the conditions of the flow-flash measurements of E278Q see [46].

## 4. Discussion

**Figure 7:**
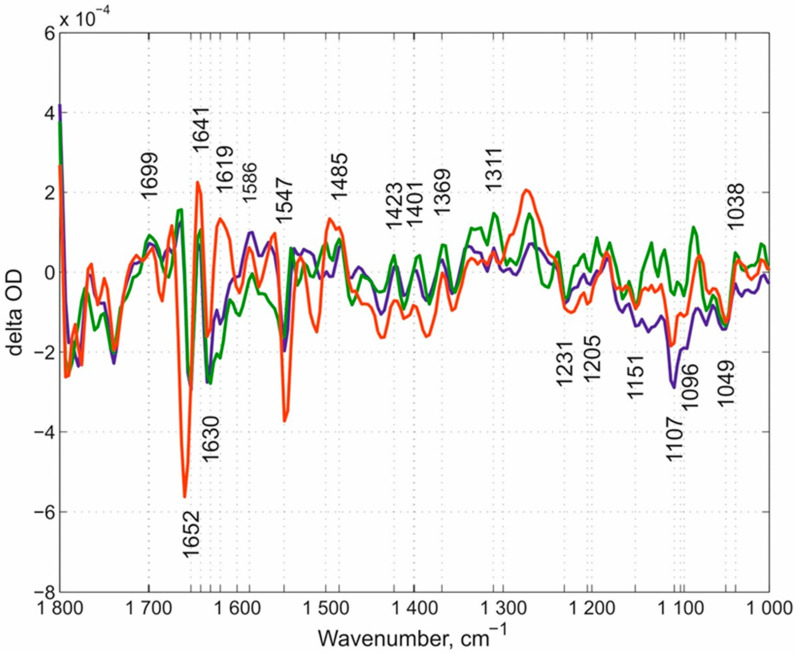
The double-difference spectra between (1) D-blocking channel mutants and (2) E278Q. The difference bands between all three cases in (i) and (ii). The double-difference bands that are present in all (i) cases and absent in (ii) are labeled plus additionally labeled bands that are seen in the double difference spectra in all three cases. N131V pH 9.0-*minus*-E278Q pH 6.5 is shown in dark blue, N131V pH 6.5-*minus*-E278Q pH 6.5 in green, and D124N pH 9.0-*minus*-E278Q pH 6.5 in red. This figure should include a signature of PLS protonation-*minus*-deprotonation.

Based on our results we discuss the potential role of different proton-loading site participants. One clear IR band is present in the “fast” kinetic spectra of N131V at both pH values and D124N mutants at ~1699 cm^−1^ position. In the E278Q enzyme, this band also appears though with a reduced intensity and is within the noise level. Despite this reduced insensitivity in the difference spectrum of the E278Q mutant within this frequency region, the observed weak intensity differences should not be neglected. To better visualize the other differences between (i) the “fast” kinetic spectra of two mutants at three conditions altogether (with loaded PLS), namely N131V at pH 9.0 and 6.5 and D124N at pH 9.0 and (ii) the “fast” kinetic spectrum of the E278Q mutant (empty PLS), the difference spectra between each of (i) and (ii) were produced and presented in Figure 7 (=the double-difference spectra). Seven different IR bands were found in the “fast” phases that (1) are present in the “fast” kinetic spectra of both N131V and D124N mutants, (2) have reciprocal counterparts in their “slow” kinetic spectra, and (3) are absent in the “fast” kinetic spectra of E278Q mutant. These bands are ~1699, ~1655, 1641, ~1550, ~1481, ~1405, and ~1140 cm^−1^. To see more bands, the double-difference spectra were calculated. Additionally, bands that are not seen in the difference spectra but are visualized in the double-difference spectra are also marked (Figure 7). Some of these bands should include a protolytic transition of the PLS (protonation in the “fast” kinetic phase and deprotonation in the “slow” one).

The identification of the IR bands of the PLS begins from the least probable candidates in order to exclude them from consideration.

1. ***Tyr.*** The first such candidate is Tyr. No signature of Tyr protonation-*minus*-deprotonation [53] is seen in the double difference spectra. In addition, there are no Tyr residues close to the A-propionate site (A-Prp) in the structure [7,8,10,11,12,54].
2. ***Asp/Glu.*** The next least probable candidates are Asp and Glu residues. Even though there is Asp-399 hydrogen bonded to A-Prp, this residue is less probable because of the position of the C=O symmetrical protonation bands for the region of 1712–1788 cm^−1^ for Asp. A similar situation is with Asp, whose band position is in the region 1716–1788 cm^−1^ for the inside of a protein [55]. There are no bands in this region in the double-difference spectra except the earlier assigned Glu-278 bands [45], seen here as a drop at ~1740 cm^−1^. Even though Asp-399 was proposed as a possible PLS in [19], no full Asp protonation signature is evident in the spectra. A recent study with mutated Asp-399 and His-433 [56] described the importance of Asp-399 together with His-433 in proton-pumping, based on their mutations. Asp-399 was shown to be important in proton-pumping [19].
3. ***Lys.*** Although the Lys side chain amino group gives rise to only weak IR bands, it can be excluded from consideration because there is no Lys close to A-Prp.
4. ***Arg.*** The following least probable candidate is Arg. Arg contribution is possible to the band at 1666 cm^−1^ (our unpublished data on Arg protolytic transition in solution) being close to that reported in [55]. Again, no Arg is found close to A-Prp in the structures [7,8,10,11,12].
5. ***Heme a_3_.*** The first probable candidate for the PLS is A-Prp of heme *a_3_* itself. Protonated propionate has an intensive absorbance at about 1700 cm^−1^ (see Internet free databases [57] for example). However, if we assume that the extinction coefficient of Prp is close to that of Glu, if it takes a proton during the “fast” kinetic phase, it must hold only a fraction of it. Another fraction in this case must be distributed between other possible protonation sites. Behr et al. published [58] the IR difference spectrum of Prp in C*c*O, but it was done for the redox static spectrum of C*c*O.
6. ***His.*** The second probable candidate is His, with both the first (imidazolium-*minus*-imidazole) and the second (imidazole-*minus*-imidazolate) protolytic transitions (for the spectra see [53]). The IR window around 1100 cm^−1^ is free from all other vibrations in enzymes except for histidines. There are two major bands that could be assigned: 1096 cm^−1^ to the 1^st^ His transition and 1107 cm^−1^ to the second transition. The assignment of other characteristic bands to His in the double-difference spectra is difficult because of their interference with other residue, amide and heme bands. There is no known data for extinction coefficients for these two bands, thus it is impossible to estimate the contribution of each of them to the double-difference spectra. Moreover, there is His-403 (Figure 1) close to A-Prp and a hydrogen bond that additionally proves that there is a contribution of His absorption to the PLS. The structural analysis of the corresponding structure (PDB ID: 3HB3, 2.25 Å) suggests that proton-pumping does not require either a histidine or an aspartate hydrogen bonded to A-Prp.
7. ***Water clusters.*** Finally, the last and the most probable candidate for consideration as a contributor to the PLS absorption is water cluster(s). Since protonated water clusters could absorb in the region 1800–1000 cm^−1^ with weak extinction [59], the observed absorption could be putatively assigned to the contribution to the protonated PLS water molecules. Supekar et al. showed using multi-scale molecular simulation that a transient PLS may indeed be a protonated water cluster in the form of a Zundel or an Eigen ion [60]. Furthermore, Lu et al. predicted from their calculations that the PLS is not simply one residue, but a cluster composed of polar residues and water molecules [61], which is in agreement with the suggestion made in our work. Finally, a number of experimental studies conducted by Palese support our hypothesis about the significance of the protonated water clusters in the realization of the proton pathway through the cytochrome C*c*O [62,63,64]. Due to the technical challenges in the assignment of the spectra of particular water molecules belonging to the “water region” (Figure 2) and because it is beyond the scope of this work, we leave the question about the roles of individual water molecules in proton transfer open for future studies. Instead we define the “water region” by the distance cut-off of 4 Å from the amino acid residues comprising the channels, shown in Figure 2.

## 5. Conclusions

In this paper, for the first time, an attempt to probe the protonation/deprotonation of the PLS of C*c*O enzyme using FTIR time-resolved spectroscopy is presented. To measure the IR spectrum of the PLS of an enzyme, a special setup was built based on a FTIR spectrometer IFS 66/s with a fast MCT detector and an ATR cell. A special gas-tight chamber with connectors to a bottle with “working buffer” was assembled on top of the ATR cell, which allowed production of the **FRCO** complex and, thus, measurement of the oxygen reaction in the selected mutated C*c*O variants. In order to attribute IR bands to protonation of the PLS, they should fulfill the following criteria: (1) be present in the “fast” kinetic spectra for N131V and D124N, (2) be reciprocal in the “slow” kinetic spectra for these mutants, and (3) be absent in the “fast” kinetic spectra of the E278Q mutant. Seven IR bands were found that satisfy these three requirements. To more accurately resolve the bands that belong to protonation/deprotonation of the PLS, the double-difference spectra between the “fast” kinetic spectra of N131V and D124N and between E278Q mutants were taken. More clear bands appeared in the so-called double-difference spectra. Some of these IR bands could be assigned partially to a Prp and also to a His residue. The Prp is most likely heme *a_3_* A-Prp, whereas the His is possibly located nearby heme *a_3_* A-Prp His-403. Water clusters absorb weakly in the studied region and could not be seen due to much stronger bands of hemes and amino acid residues. Nevertheless, water cluster(s) is/are possible candidate(s) for the PLS and should be expected in the “water region” with a small extinction. To sum up, we propose a molecular mechanism of proton-loading in the enzyme C*c*O by applying FTIR techniques. Besides deepening the understanding of the molecular basis of this particular enzyme function, the methodological pipeline established and used in this work represents a promising experimental approach that could be further adapted to successfully characterize other protein-transporting enzymatic systems.

## Figures and Tables

**Table 1:** Summary of the assignments of the bands mentioned through the manuscript.

Wavenumber, cm^−1^	Assignment
1965	C≡O-heme *a_3_*
1712–1788	Asp-399
1740	Glu-278
1700	A-Prp of heme *a_3_*
1666	Arg residues
1308	Tyr-280
1247	Tyr-35
1107	His residues, second transition
1096	His residues, first transition
1000–1800	Water

## Abbreviations

ATR	attenuated total reflectance
BG	background
BNC	binuclear center
C*c*O	cytochrome *c* oxidase
FRCO	fully-reduced CO-inhibited enzyme
FTIR	Fourier transform infrared
IR	infrared
PLS	proton-loading site
Prp	propionate
